# Case Report: Diffuse Lumbar Hyperostosis Causing Vertebral Canal Stenosis in a Dog With Concurrent Multicentric T-Cell Lymphoma

**DOI:** 10.3389/fvets.2022.825525

**Published:** 2022-06-22

**Authors:** Max Foreman, Audrey Belmudes, Elizabeth Villiers, Elena Scarpante

**Affiliations:** ^1^Neurology and Neurosurgery Service, Dick White Referrals, Part of Linnaeus Veterinary Limited, Cambridgeshire, United Kingdom; ^2^Diagnostic Imaging Service, Dick White Referrals, Part of Linnaeus Veterinary Limited, Cambridgeshire, United Kingdom; ^3^Clinical Pathology Service, Dick White Referrals, Part of Linnaeus Veterinary Limited, Cambridgeshire, United Kingdom

**Keywords:** magnetic resonance imaging (MRI), neoplasia, neurology, paraparesis, paraneoplastic

## Abstract

A 4-year-old female spayed Bullmastiff-cross presented with a 24-h history of progressive paraparesis. Neurological examination was consistent with L4–S3 myelopathy. On magnetic resonance imaging (MRI), all vertebrae showed homogenously increased short tau inversion recovery (STIR) signal with strong contrast enhancement. The vertebral canal was concentrically narrowed along the length of the L5 vertebra secondary to bony proliferation of the vertebral pedicles, dorsal lamina, and vertebral body. Cytological analysis of the peripheral lymph nodes and subsequent flow cytometry was consistent with T-cell lymphoma. The dog was euthanised due to poor prognosis. Necropsy confirmed the presence of stage V multicentric T-cell lymphoma, as well as diffuse hyperostosis of the vertebral bodies. This is the first report of presumed paraneoplastic lumbar skeletal hyperostosis.

## Introduction

Lymphoma is one of the most frequently diagnosed malignancies in the dog, with medium-sized and larger breeds being overrepresented. A familial occurrence has been reported in several breeds, including the Bullmastiff (1). The most common clinical presentation is the multicentric form affecting multiple peripheral lymph nodes, however extranodal forms exist within all other body systems. Primary osseous lymphoma is rarely reported in dogs (2). More commonly, osseous and vertebral lymphoma in dogs is diagnosed as part of a multicentric process (3–6). T-cell and stage V lymphomas carry a poorer prognosis (1) when compared to B-cell or lower stage lymphomas. T-cell lymphoma appears to be rarely associated with osseous involvement, with only one out of a 46 cases of T-cell lymphoma involving bone in one study (7). Spinal extradural T-cell lymphoma has also been reported in dogs, however there was no evidence of bony infiltration in these cases (8, 9).

Osseous lymphoma in dogs may be radiographically silent (6, 10), or may show a variety of non-specific radiological changes, most commonly osetolysis (2, 4, 10). Diffuse smooth diaphyseal periosteal reaction was seen in the long bones of one dog with polyostotic lymphoma (4), and irregular osteoproductive lesions were present associated with the vertebrae of another (5). Vertebral changes on MRI may be confined to the medullary cavity (3) or may extend into the extradural and paraspinal tissues (6).

Various paraneoplastic syndromes have been reported associated with canine lymphoma. Hypercalcaemia is most common (1, 11), however other conditions reported include neuropathy (12), polycythaemia (13), hypoglycaemia (14), monoclonal gammopathy (15), eosinophilia (8) and immune-mediated diseases (16). Hyperostosis can occur as a paraneoplastic condition most commonly secondary to both neoplastic and non-neoplastic pulmonary disease - hypertrophic osteopathy (HO) - and causes a painful periosteal reaction and associated soft tissue swelling of the distal limbs (17). Extrapulmonary neoplastic causes have been reported as well, including tumours of the bladder and kidney. (18–21). In human medicine, axial skeletal hyperostosis has been reported in a case of systemic Hodgkin's lymphoma (18).

To the authors' knowledge, a hyperostotic syndrome has not been reported in association with canine lymphoma. Therefore, this case report describes the presence of an unusual distribution of hyperostosis in a dog concurrently affected by atypical multicentric T-cell lymphoma.

## Case Description

A 4-year-old female spayed Bullmastiff-cross was referred with a 24-h history of progressive paraparesis. The dog was reported to have had a two-month history of intermittent vomiting and frequent diarrhoea, weight loss of 2 kilograms and more recently suspected polyuria-polydipsia - the referring veterinary surgeon had diagnosed hypocobalaminaemia and initiated supplementation. Subsequently, the dog had become progressively unable to stand on the pelvic limbs.

General physical examination documented poor body condition, generalised peripheral lymphadenomegaly affecting the mandibular, superficial cervical and popliteal lymph nodes, and instability of the right stifle. On neurological examination, the patient was ambulatory paraparetic with reduced pelvic limb, tail and anal tone, reduced patella and pelvic limb withdrawal reflexes and absent pelvic limb proprioception. Focal lumbar pain was present. Neuroanatomical localisation was consistent with L4–S3 myelopathy, with differentials for the paraparesis considered including degenerative, inflammatory, neoplastic and infectious diseases. Differential considered for the peripheral lymphadenomegaly included neoplastic, reactive, inflammatory and infectious diseases.

## Diagnostic Investigations and Outcome

Complete blood count, biochemistry and electrolyte profiles were unremarkable, however a moderate proportion of lymphocytes appeared reactive on smear examination. Total calcium was within the reference range, hence ionised calcium was not assessed. C-Reactive Protein was elevated (21 mg/L; reference range <10). Urinalysis showed the urine to be well concentrated with a specific gravity of 1.033, trace protein and evidence of a urinary tract infection, with 10–20 white blood cells/high power field (Ref. 0–2). Urine culture was positive for *E. coli*.

Under general anaesthesia, magnetic resonance examination of the lumbosacral vertebral column was performed (Hitachi Aperto Lucente 0.4 Tesla, Berkshire, UK). Imaging was performed in dorsal recumbency with the pelvic limbs in extension. The following multiplanar MR sequences were performed: sagittal T2-weighted (T2W; TE=120 ms; TR=3290 ms; 3 mm thick with no interslice gap), transverse T2-weighted (T2W; TE=100 ms; TR=5653 ms; 4 mm thick with no interslice gap), sagittal short-tau inversion recovery (STIR; TE=60 ms; TR=3014 ms; 3 mm thick with no interslice gap), sagittal and transverse T1-weighted (T1W; TE=13 ms; TR=745 ms; 3 mm and 4 mm thick respectively with no interslice gap). Following intravenous administration of 0.1 mmol/kg gadolinium-based contrast agent (Dotarem, Guerbet LLC, Ohio, USA), sagittal and transverse T1-weighted sequences were obtained (T1W; TE=13 ms; TR=745 ms; 3 mm and 4 mm thick respectively with no interslice gap). Sagittal sequences were acquired from L2 to Cd4, and transverse sequences acquired from L4–5 to L6. Transverse T2-weighted sequence was also acquired across L7–S1.

**Figure 1:**
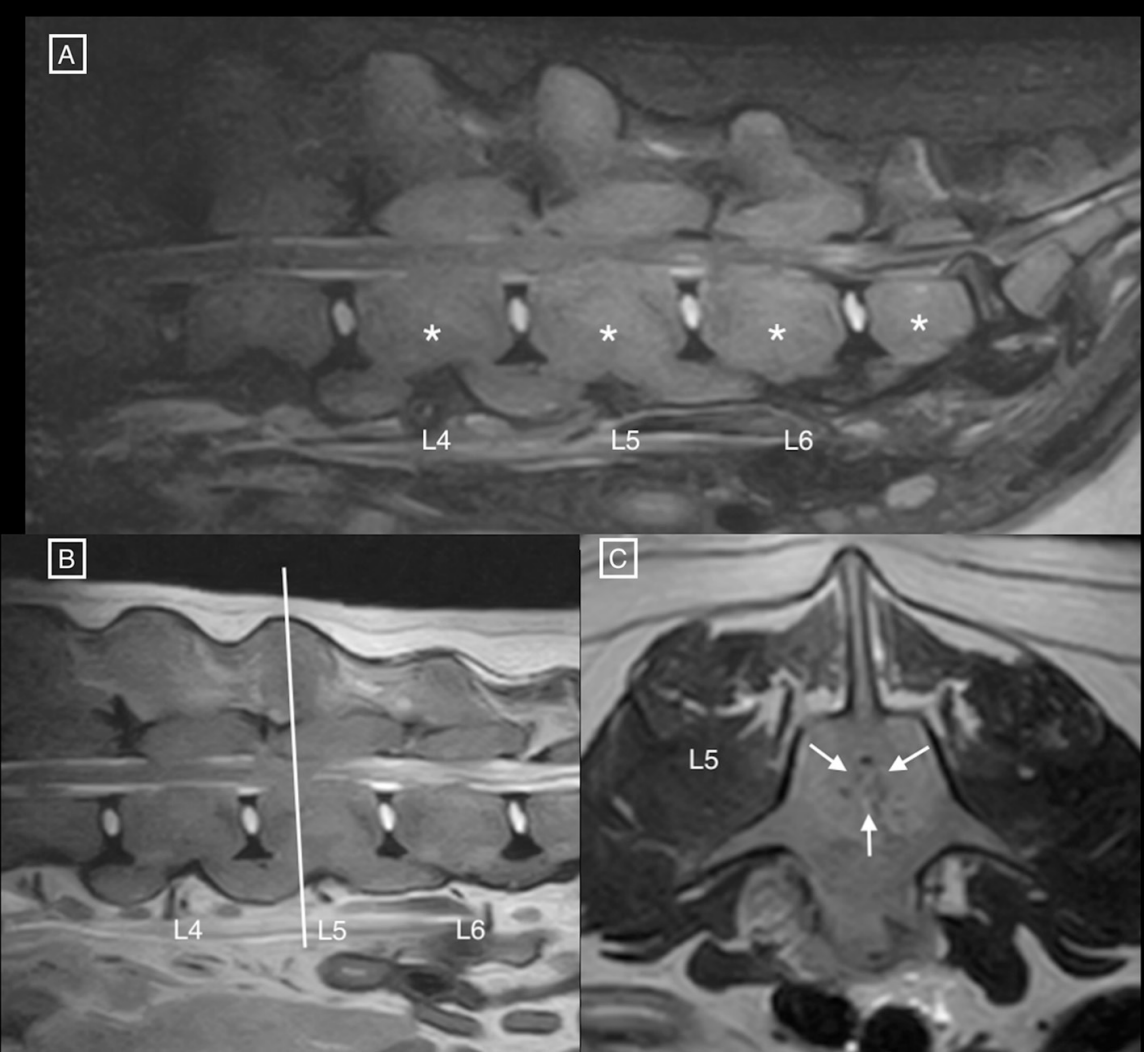
**(A)** Sagittal STIR and **(B)** T2 MR images of the lumbar spine, and **(C)** transverse T2 MR image at the level of mid-L5. Notice the diffuse hyperintensity of the vertebral structures (asterisks), associated with circumferential concentric bone thickening, and secondary narrowing of the vertebral canal (white arrows) leading to circumferential compression of the spinal cord. The nucleus pulposus is also decreased in intensity at L7–S1, and the annulus fibrosus protrudes dorsally in the vertebral canal.

**Figure 2:**
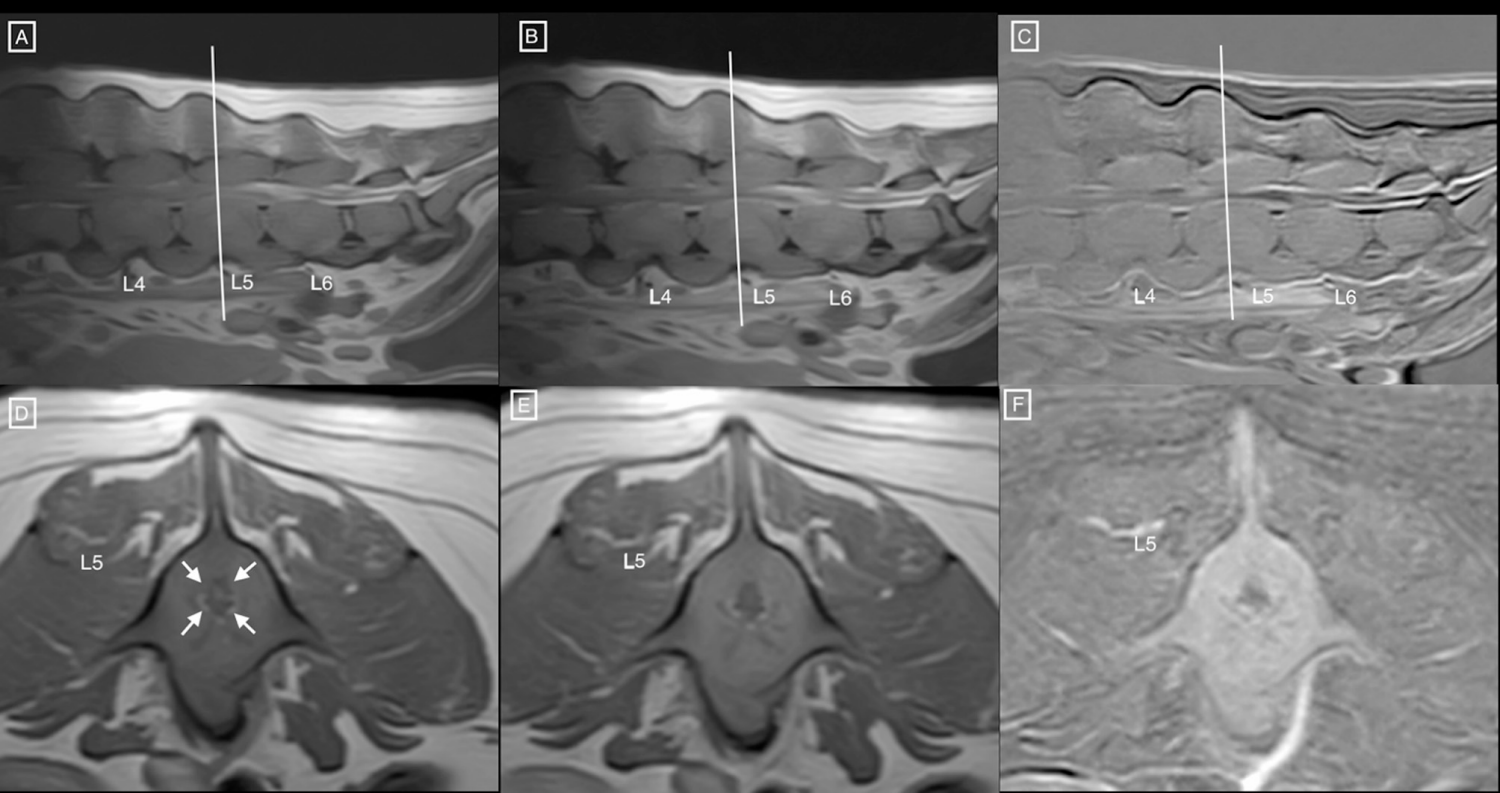
Sagittal T1 weighted **(A)** pre and **(B)** post contrast MR images of the lumbar spine, and **(C)** corresponding subtraction image. **(D)** Transverse T1 weighted pre and **(E)** post contrast MR images at the level of mid-L5 and **(F)** corresponding subtraction image. Notice the diffuse hyperintensity, homogeneous enhancement of the vertebral structures, associated with circumferential concentric bone thickening, and secondary narrowing of the vertebral canal (white arrows) leading to circumferential compression of the spinal cord. Similar changes are noted at L4 and L6.

Diffusely abnormal bone signal was observed in all visible vertebrae, showing an increase in STIR signal and normal to increased T2 signal (Figure 1) with diffuse and homogenous contrast enhancement (Figure 2). The signal intensity changes were associated with circumferential concentric thickening of the vertebral pedicles, dorsal lamina and vertebral body, leading to marked circumferential narrowing of the vertebral canal along the length of L5 with secondary spinal cord compression. There was complete circumferential attenuation of the CSF signal surrounding the spinal cord at this level (Figure 1). There were similar but less marked changes affecting the L4 and L6 vertebrae. The normal shape of the vertebral bodies was maintained, with persistent visualisation of the external cortical bone but loss of visualisation of the inner cortex. Moderate flowing ventral ossification was also noted along the length of the lumbar spine, bridging the vertebral bodies from L3 to L7 with absence of extensive changes of degenerative disc disease. Additionally, moderate bilateral medial iliac lymphadenomegaly was noted. Differential diagnoses considered for concentric osseous thickening included generalised osteoproliferative disease (osteochondromatosis, atypical hypertrophic osteopathy), myeloproliferative disorders (myelofibrosis), osteopetrosis, hypervitaminosis D, metabolic disease (hypoparathyroidism, hypervitaminosis A) or less likely neoplastic infiltration (round cell neoplasia). The changes noted along the ventral aspect of the lumbar spine were consistent with diffuse idiopathic spinal hyperostosis (DISH).

**Figure 3:**
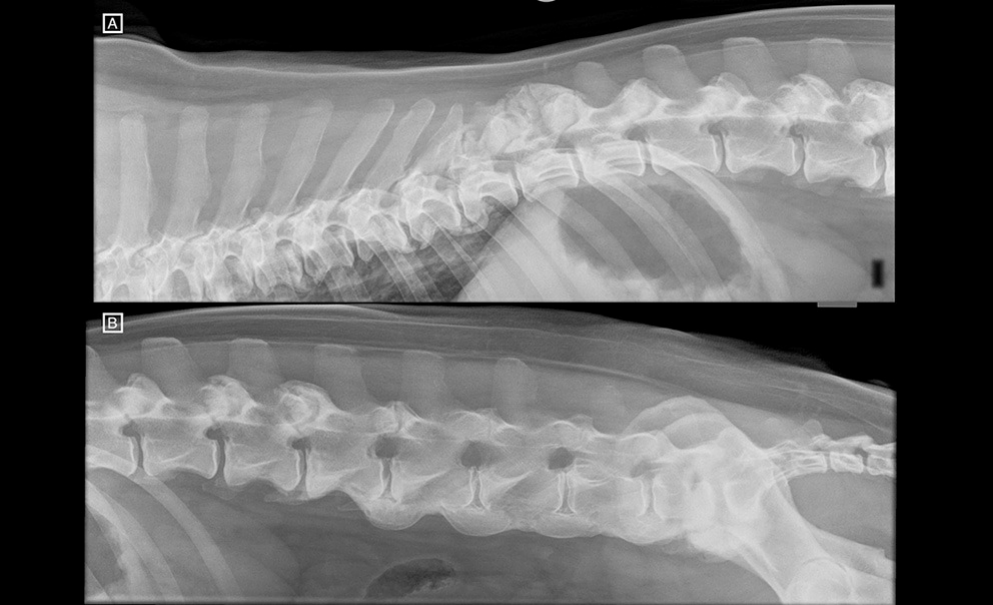
**(A)** Right lateral radiographs of the thoracolumbar and **(B)** lumbosacral spine. T13 has the last pair of ribs. Notice the new bone formation around the spinous process of T11 and T12 and the articular processes at T11–12 and T12–13. The lumbar vertebrae appear decreased in radiopacity, and the dorsal cortex of the vertebral bodies is not identified. There is ventral bridging spondylosis along the ventral aspect of the lumbar spine, from L3 to LS (ventrally to the endplates but extending ventrally to the vertebral body as well) causing a flowing ossicification consistent with diffuse idiopathic skeletal hyperostosis (DISH). There is further ventral bridging spondylosis at the level of T7–T8 and T9–T11.

Right lateral radiographs of the vertebral column from T6 to Cd2 (Figure 3) showed normal to decreased bone radiopacity of the lumbar vertebrae, and loss of visualisation of the dorsal cortex of the vertebral body from L1 to L6. Moderate new bone formation around the spinous processes of T11 and T12, and the articular facets at the level of T11–12 and T12–13, and more mildly at L1–2 and L2–3 was noted. Furthermore, the same flowing ossification along the ventral aspects of the vertebral bodies from L3–L7 was noted, as well as ventral bridging spondylosis at the levels of T9–11, T7–8 and L7–S1.

Bone biopsy of the new bone around the vertebral articular facets at T11–13 consisted of mature cartilage and bone, which was disorganised and forming thick trabeculae. There was no evidence of inflammation or malignant neoplasia.

Fine needle aspiration of the popliteal lymph nodes showed significant expansion in the proportion of intermediate-sized lymphoid cells which were approximately 1.5 times the diameter of small lymphocytes. They had a round and slightly irregular shaped nucleus, slightly dispersed chromatin but no nucleoli, and a small to moderate amount of pale-staining cytoplasm. The cytological diagnosis was most suggestive of lymphoma although an atypical hyperplasia was not excluded. Flow cytometry was performed, using leukocyte marker CD45, B-cell markers CD21 and CD79a, T-cell markers CD3 and CD5, T-helper cell marker CD4, cytotoxic T-cell marker CD8, early precursor marker CD34 as well as MHC II. The cells assessed were CD45 positive in 99%, with 98% positive for CD3 and 21% positive for CD4. The cells were negative for CD5 and MHC II. This atypical immunophenotype (CD3+ / CD5- / variable CD4) confirmed a T-cell lymphoma and excluded hyperplasia.

**Figure 4:**
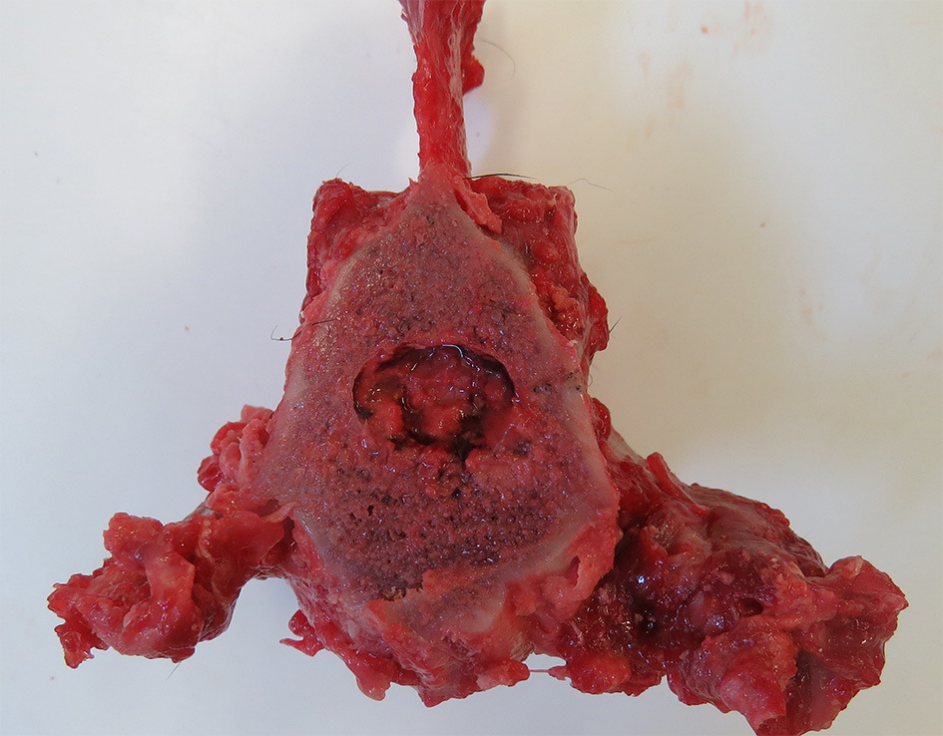
Post-mortem specimen; transverse section at the level of mid-L5 vertebral body. Note the concentric narrowing of the vertebral canal, as well as the hyperostosis and smooth osseous proliferation of the articular surfaces.

The owner elected for euthanasia. On gross necropsy the lumbar vertebrae were thickened (hyperostosis), with the articular surfaces covered in smooth osseous proliferation (Figure 4). Following sectioning, narrowing of the spinal canal was seen, and the vertebral bodies of the L4–6 vertebrae were enlarged. Additionally, a multinodular proliferation was present, adhered to the left cranial dorsal lung and expanding the mediastinal space, the liver showed a diffuse lobular pattern, the spleen was diffusely enlarged, and the mesenteric and retroperitoneal lymph nodes were enlarged.

All major body organs were examined histopathologically. Proliferations of densely cellular, infiltrative round cells were present in the thymus, liver, spleen, lymph nodes and gastric wall. These neoplastic cells were arranged in sheets on a fine fibrovascular stroma, were approximately 1–1.5xRBC, round with distinct cell borders and scant eosinophilic cytoplasm. Nuclei were round to oval with coarsely stippled chromatin. There was mild anisocytosis and anisokaryosis and marked multifocal necrosis. Frequently neoplastic cells were seen migrating across vascular adventitial layers. The bone marrow of L5 was diffusely expanded and hypercellular and was infiltrated with the same monomorphic population of monocytic cells. Multifocally within the epidural adipose tissue was a similar mononuclear infiltrate. Smaller clusters of rounds cells appeared next to nerve roots within the intradural space (intradural, extramedullary). No neoplastic invasion of the hyperostotic cortical bone was noted. The necroscopic findings were consistent with stage V multicentric lymphoma with concurrent vertebral hyperostosis. The cell size, lack of auxiliary plasma cells and amyloid production was consistent with T-cell differentiation, as suspected from the flow cytometry.

## Discussion

T-cell lymphoma (TCL) represents around 30–40% of canine lymphomas, with prevalence varying among breeds (22). Most TCL are peripheral, with the nodal form being the most common (23). The case presented here had a typical multicentric distribution for a TCL, with the mediastinal involvement having also been associated with the T-cell phenotype (24). The flow cytometry performed was atypical for a TCL, as the cells were positive for the CD3 T-cell marker, but negative for CD5 T-cell marker and MCH II. The flow cytometry was repeated in this case to confirm the immunotype. Among peripheral TCLs, MHC II loss is frequent. Loss of CD5 is reported to aberrantly occur and may have a prognostic Implication (25).

The medullary cavity changes on MRI in the case reported here are consistent with those previously described (3), being T1 isointense, T2 iso to hyperintense and STIR hyperintense, and strongly contrast enhancing. These findings are compatible with replacement of the medullary bone fat with malignant cellular infiltrate. What is novel about this case is the presence of diffuse, bilateral and symmetrical periosteal new bone formation affecting the lumbar vertebrae in the absence of osteolysis or focal vertebral lesions. Most unusually, this hyperostosis caused circumferential narrowing of the vertebral canal and secondary spinal cord compression, which to the authors' knowledge has not previously been reported. The changes however appear analogous to a human case report of meningeal Hodgkins lymphoma, with adjacent calvarial hyperostosis without histological evidence of tumour cell invasion (18). It is not clear in this case whether the rapid onset of neurological signs relates to the bony compression, or otherwise due to the concurrent infiltration of the epidural and intradural spaces compounding the spinal cord compression.

Hypertrophic osteopathy is a well characterised syndrome in humans and animals that may develop in response to neoplastic and non-neoplastic pulmonary disease, as well as in extra-pulmonary neoplastic disease (17–21). Despite its frequency, the underlying pathogenesis is still not well understood. Recently, most attention has been given to the role of vascular endothelial growth factor (VEGF) and platelet-derived growth factor (PDGF) in this condition, with over-expression and activation inducing the stromal and vascular changes seen in HO (26). Both VEGF and PDGF overexpression has been associated with non-Hodgkin's lymphoma in humans (27, 28). It is therefore possible that the case presented here represents a novel form of paraneoplastic HO, with the changes instead affecting the axial skeleton. Imaging of the distal appendicular skeleton was not performed in this case, and the long bones were not examined during necropsy. Despite there being no clinical findings to suggest it, concurrent typical changes of HO in the distal appendicular skeleton cannot be ruled out.

In addition to the diffuse enlargement and hyperostosis affecting the vertebral bones of L4–6, both MRI and radiographic imaging of the spine demonstrated changes typical of diffuse idiopathic skeletal hyperostosis (DISH). These include calcification and ossification along the ventral aspects of three contiguous vertebral bodies, absence of extensive radiographic changes of degenerative disc disease, peri-articular osteophytes around vertebral joints and calcification and ossification of soft tissue attachments in both the axial and peripheral skeleton (19). Therefore, the mature cartilage and bone proliferation found around the articular facets of the thoracic vertebrae were considered a component of DISH rather than part of the proposed paraneoplastic syndrome. DISH is a common non-inflammatory systemic disease of the spine and abaxial skeleton of dogs, which is often incidental although can rarely be associated with clinical signs (20–22). Hyperostosis of the internal vertebral cortical bone causing vertebral canal narrowing is not reported in DISH, however it is possible that all of the bony changes reported here may represent a novel presentation of DISH that is unrelated to the lymphoma.

To the authors' knowledge, this is the first reported case of suspected paraneoplastic hyperostosis in the axial skeleton of a dog with confirmed stage V T-cell lymphoma, leading to circumferential spinal cord compression.

## Data Availability Statement

The original contributions presented in the study are included in the article/supplementary material, further inquiries can be directed to the corresponding author/s.

## Ethics Statement

Ethical review and approval was not required for the animal study because this is a retrospective clinical case report. Written informed consent was obtained from the owners for the participation of their animals in this study.

## Author Contributions

Conception and design: MF and ES. Acquisition of data, analysis and interpretation of data, revising article for intellectual content, and final approval of the completed article: MF, AB, EV, and ES. Drafting the article: MF. All authors contributed to the article and approved the submitted version.

## Funding

Funding for this publication was provided by Linnaeus Veterinary Limited. The funder was not involved in the study design, collection, analysis, interpretation of data, the writing of this article or the decision to submit it for publication.

## Conflict of Interest

All authors were employed by company Linnaeus Veterinary Limited.

## Publisher's Note

All claims expressed in this article are solely those of the authors and do not necessarily represent those of their affiliated organizations, or those of the publisher, the editors and the reviewers. Any product that may be evaluated in this article, or claim that may be made by its manufacturer, is not guaranteed or endorsed by the publisher.
